# Transition nutritionnelle, prévalence de la double charge de la malnutrition et facteurs de risque cardiovasculaires chez les adultes de l'île comorienne d'Anjouan

**DOI:** 10.11604/pamj.2020.35.89.19043

**Published:** 2020-03-25

**Authors:** Rachmat Attoumane Ben Ali, Khouloud Harraqui, Zineb Hannoun, Mouhidine Monir, Mohamed Samir, Mohamed Anssoufouddine, Abdellatif Bour

**Affiliations:** 1 Laboratoire des Essais Biologiques, Equipe de Transition Alimentaire et Nutritionnelle, Faculté des Sciences, Université Ibn Tofail, BP 133, Kenitra 14000, Morocco; 2 Unité de Recherche de Décentralisation et Cohésion Sociale, Faculté de Droit et des Sciences Economiques, Université des Comores, BP 2585 Moroni, Union des Comores; 3 Service Médical, Centre Hospitalier Régional d'Anjouan, BP 23 Mutsamudu, Union des Comores

**Keywords:** Prévalence, insuffisance pondérale, surpoids, obésité, facteurs de risque cardiovasculaires, Anjouan, Comores, Prevalence, underweight, overweight, obesity, cardiovascular risk factors, Anjouan, Comoros

## Abstract

**Introduction:**

le surpoids et de l'obésité progressent de manière effrayante, notamment dans les pays en développement. Cette étude vise à déterminer la prévalence de l'insuffisance et de la surcharge pondérale et à évaluer la relation entre l'Indice de Masse Corporelle et les facteurs de risque cardiovasculaires associés chez les adultes de l'île d'Anjouan.

**Méthodes:**

l'enquête est une étude transversale, où un échantillon de 902 individus âgés de 25 à 64 ans est sélectionné en utilisant la méthode de sondage empirique « des quotas ». Le statut nutritionnel est déterminé en calculant l'indice de masse corporelle (IMC), le périmètre abdominal et le rapport tour de taille/tour de hanche (RTH). La pression artérielle, le périmètre abdominal et le tour de hanche ont été mesurés pendant l'interview tandis que la glycémie capillaire à jeun a été mesurée le lendemain.

**Résultats:**

les résultats ressortis font état d'une moyenne d'âge de 39,5 ± 11,67 ans. La prévalence globale de l'insuffisance pondérale, du surpoids et de l'obésité est respectivement de 4,1%, 28,6% et 22,2%. Les facteurs de risque associés au surpoids/obésité sont l'âge avancé (P= 0,004), le genre (P=0,000), le poids (P=0,000), le diabète (P= 0,006), l'hypertension (P= 0,01), l'obésité abdominale (P= 0,000), le tour de hanche (P=0,000), le RTH (P=0,000), la durée inactive/jour (P=0,001) et le tabagisme (P< 0,05), contrairement à l'inactivité physique (P= 0,10).

**Conclusion:**

les résultats confirment la présence du double fardeau nutritionnel. D'où l'urgence de mettre en place des stratégies de prévention des maladies non transmissibles.

## Abstract (en)

**Introduction:**

Overweight and obesity has been increasing at an alarming rate, in particular in the developing countries. The purpose of this study was to determine the prevalence of underweight and overweight and to assess the relationship between body mass index and cardiovascular risk factors in adults living in the island of Anjouan.

**Methods:**

We conducted a cross-sectional study involving a sample of 902 individuals aged 25 to 64 years using the empirical survey based on “quota sampling”. Nutritional status was determined by calculating the body mass index, the abdominal circumference and waist-to-hip ratio (WHR). Blood pressure, abdominal circumference and hip circumference were measured during the interview while capillary fasting blood glucose was measured the next day.

**Results:**

The results show an average age of 39.5 Â± 11.67 years. The overall prevalence of underweight, overweight and obesity was 4.1%, 28.6% and 22.2% respectively. The risk factors associated with overweight/obesity were: advanced age (p= 0.004), gender (p=0.000), weight (p=0.000), diabetes (p= 0.006), hypertension (p= 0.01), abdominal obesity (p= 0.000), hip circumference (p=0.000), WHR (p=0.000), time of inactivity/day (p=0.001) and smoking (p< 0.05), in contrast to physical inactivity (p= 0.10).

**Conclusion:**

These results confirm the presence of a double burden of nutrition. Hence the urgency to implement preventive measures for noncommunicable diseases.

## Introduction

A la différence des pays industrialisés, où la transition épidémiologique a été marquée par l'émergence des niveaux de maladie non transmissible et accompagnée d'une chute marquée dans la morbidité, des maladies infectieuses et de la mortalité [1], les pays du Sud, depuis une trentaine d'années, font non seulement face aux maladies chroniques [2] telles que, l'hypertension et le diabète mais en plus cumulent ces dernières aux pathologies carentielles et infectieuses déjà présentes [3]. Ces changements dans le profil des maladies, habituellement rencontrées dans les pays en développement, sont associés à des changements de comportement, de mode de vie, d'alimentation, du manque d'activité physique, du tabagisme et de la consommation d'alcool [4]. L'Organisation Mondiale de la Santé (OMS) estime que les Maladies Non Transmissibles (MNT) sont responsables de 60% des décès et 47% de la charge mondiale de morbidité [5]. Le surpoids et l'obésité sont des facteurs majeurs dans l'apparition du syndrome métabolique, du diabète, de l'hypertension, des maladies cardiovasculaires et des problèmes rhumatologiques [6-8], l'OMS parle d'épidémie mondiale [9]. La Fédération mondiale de l'obésité estime qu'en 2010, on compterait plus d'un milliard d'adultes en surpoids et 475 millions d'obèses dans le monde [10]. L'Union des Comores, Petit Etat Insulaire en Développement (PEID), composée d'environ 763 952 habitants [11], est en phase de transition épidémiologique. Cette dernière est marquée par des maladies transmissibles et non transmissibles, avec prédominance chez les enfants de moins de 5 ans, des maladies diarrhéiques et des Infections Respiratoires Aiguës (IRA), aggravées par la malnutrition et une augmentation des maladies chroniques [11]. En 2011, l'enquête STEPS sur les facteurs de risque des maladies non transmissibles a montré que 39,4% des adultes ont un indice de masse corporelle supérieur ou égal à 25 kg/m² dont 25,9% sont en surcharge pondérale; 13,5% sont obèses; 25,4% ont une tension artérielle élevée dont 86% ne suivent aucun traitement médical et que 61,7% d'entre eux ne pratiquent aucune activité physique régulière [11,12]. La coexistence de la sous-nutrition et de la surnutrition caractérise la transition nutritionnelle et une double charge nutritionnelle [4] dont souffrent les pays en développement. Cette étude prévoit d'estimer la prévalence de l'insuffisance pondérale, du surpoids et de l'obésité et d'évaluer la relation entre l'indice de masse corporelle et les facteurs de risque cardiovasculaires associés chez les adultes de l'île d'Anjouan (Comores) et ainsi déterminer la présence ou non de la double charge nutritionnelle.

## Méthodes

**Sélection des individus**: cette étude transversale, descriptive et analytique est réalisée sur l'île comorienne d'Anjouan, du 16 juillet au 25 août 2017. La population cible est composée d'adultes âgés de 25 à 64 ans, à l'exclusion des femmes enceintes. L'échantillon théorique, représentatif de la population, est déterminé à l'aide de la formule de la précision de l'estimation de l'intervalle de confiance de la prévalence du diabète sucré et en se basant sur les prévisions du Recensement Général de la Population et de l'Habitat (RGPH) réalisé aux Comores en 2003 [13], l'échantillon est de 864. Pour ce faire, il est considéré une marge d'erreur de 2% et un niveau de confiance de 95%. Ensuite, cette taille théorique est multipliée par l'inverse du taux de participation estimé à 80%, sur la base des enquêtes organisées à Anjouan dans le domaine de la santé ; ce qui a donné une taille d'échantillon estimée à 1080 sujets. La sélection des individus de l'échantillon est faite suivant la méthode de sondage empirique des quotas avec comme critères de quota, le sexe et l'âge. En effet, cette méthode des quotas, choisie en raison de l'indisponibilité d'une base de sondage, a consisté à sélectionner un échantillon dont la structure par sexe et par âge est très proche de celle de la population cible. Dans la mise en œuvre de cette méthode, nous avons sélectionné, de façon aléatoire avec l'aide de la procédure de « Randomisation » en utilisant la fonction ALEA du logiciel Ms Excel, 20% de l'ensemble des 91 localités de l'île d'Anjouan. Ceci a donné 20 localités périurbaines d'Anjouan sélectionnées aléatoirement, donc de façon représentative. Ensuite, les ménages à enquêter sont sélectionnés, sur le terrain, en suivant les quotas préétablis suivant les deux critères, sexe et âge. Ainsi, les ménages sont sélectionnés au hasard, en suivant la direction de la pointe d'un stylo, ce qui a garanti la représentativité des ménages sélectionnés. Au sein du ménage échantillonné, l'individu enquêté est sélectionné à l'aide de la méthode de KISH [14], ce qui donne la garantie de la représentativité des individus enquêtés. Après avoir obtenu le consentement éclairé du sujet, ce dernier est soumis au questionnaire. Un sujet est déclaré absent après 2 visites ou s'il voyageait.

**L'état nutritionnel**: est déterminé selon les critères de l'OMS [14] (IMC = poids (kg)/ taille(m^2^) correspondant à : 1) Etat dénutris si IMC <18,5 kg/m^2^; 2) Sujet sain si 18,5 < IMC <24,9 kg/m^2^; 3) Sujet en surpoids si 25 < IMC < 29,9 kg/m^2^; 4) Sujet obèses IMC >30 kg/m^2^. L'obésité abdominale est estimée par le tour de taille (≥ 80cm pour les femmes et ≥ 94 cm pour les hommes) et le rapport tour de taille / tour de hanche (RTH) (≥ 0,81 pour les femmes et ≥ 0,96 pour les hommes). Selon les normes internationales [15], le sujet est considéré comme diabétique s'il a une glycémie capillaire à jeun supérieure ou égale à 1,26 g/l ou s'il est diagnostiqué par un professionnel de la santé et il est prédiabétique si la glycémie capillaire à jeun est comprise entre 1,10 et 1,25 g/l. La pression artérielle (PA) est mesurée à l'aide d'un tensiomètre TORM BRAS BP 3NZ1-3P, en position assise, à l'aide d'un brassard bras et de taille appropriée après une période de repos de 5 minutes. Le patient est considéré comme hypertendu s'il est traité pour une hypertension connue ou si sa pression artérielle systolique moyenne est supérieure ou égale à 140 mmHg et/ou sa pression artérielle diastolique moyenne est supérieure ou égale à 90 mmHg. L'inactivité physique est évaluée par une série de questions inspirées de la littérature scientifique [16]. La consommation de tabac, d'alcool et le style de vie sont déterminés via un questionnaire préparé à cet effet. Par ailleurs dans le cadre de ce même projet nous avons utilisé un questionnaire de fréquence alimentaire (FFQ ) adapté aux coutumes alimentaires du pays, afin d'évaluer les habitudes alimentaires des sujets enquêtés.

**L'analyse statistique**: est faite suivant le protocole suivant: les données collectées sont saisies sur Excel. Après filtration et codification, elles sont transmises sur un support d'exploitation statistique. Les résultats sont exprimés par la suite, sous forme de moyenne ± écart type, ou sous forme de pourcentage. Les tests d'hypothèses à 5% d'erreur appliqués pour comparer les moyennes ou les proportions sont respectivement le test T de student ou le test d'indépendance Khi-2.

## Résultats

**Figure 1:**
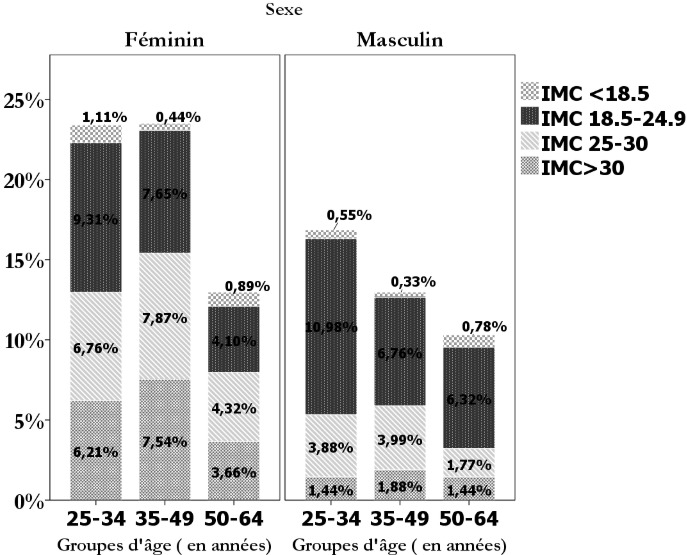
Distribution de l'indice de masse corporelle (kg/m^2^) de la population étudiée en fonction du genre et des groupes d'âge

L'étude est portée sur 1080 enquêtés au hasard, mais seuls 902 personnes ont répondu aux questions proposées dans la fiche d'enquête, le taux de participation s'élève donc à 83,5%. La population étudiée est composée de 540 femmes (59,9%) et 362 hommes (40,1%) avec un sex ratio de 0,67. Le tableau 1 représente la moyenne des variables étudiées. Sur les 902 participants, 37 (4,1%) individus souffrent d'insuffisance pondérale, tandis que le surpoids et de l'obésité sont retrouvés, respectivement, chez 258 (28,6%) et 200 (22,2%) participants avec une prédominance significative chez les femmes pour chaque état nutritionnel (P<0,05) (Figure 1). La maigreur touche à part égale (1,7%) les jeunes adultes et les plus de 50 ans (tous sexes confondus) tandis que le surpoids et l'obésité sont plus fréquents dans la tranche d'âge 35-49 ans (P=0,004). Quant à l'inactivité physique, la consommation du tabac et de l'alcool, on les retrouve, respectivement, chez 665 (73,7%), 95 (14,7%) et 1 (0,2%) sujets. En comparant avec le genre masculin, la gent féminine est plus inactive à raison de 398 (44,1%) contre 267 (29,6%) participants masculins, avec une différence statistiquement significative (P=0,008). En outre, 590 sujets (65,4%) témoignent rester plus de 12H/jour en position assise ou couchée dont 392 (43,5%) femmes (P<0,001). Dans le même concept, la sédentarité est plus marquée chez les jeunes adultes (29%) suivie de la tranche d'âge 35-49 ans (27,5%) et des sujets âgés (17,2%) mais sans différence statistique. La consommation du tabac concerne particulièrement les hommes (11,2% VS 3,6%) et les sujets âgés de plus de 50 ans (6,4%) contre 3,7% et 4,7%, respectivement, pour les classe d'âge 35-49 ans et 25-34 ans ; avec un gradient positif (P=0,000) pour les deux variables. Il en va de même pour l'alcool en ce qui concerne le genre.

La moyenne de l'IMC (Tableau 1) est hautement élevée chez les femmes (27,52± 6,14) que chez les hommes (24,45±4,16) (P<0,001), ainsi que l'obésité abdominale (P<0,001). Les moyennes des paramètres anthropométriques sont significativement élevés (P<0,001) chez les sujets ayant un IMC>30 kg/m^2^ par rapport aux sujets sains ou souffrant de maigreur (Tableau 2). On retrouve la même allure pour les paramètres cliniques, exception faite pour la moyenne glycémique qui est supérieur chez les sujets ayant un IMC <18,5 kg/m^2^ comparé aux sujets ayant un 18,5< IMC<30, considérés normaux (Tableau 2), mais sans différence statistique significative. La prévalence du diabète (4,8%) augmente significativement avec l'âge (P<0,001), toutefois aucune différence statistique n'est observée en ce qui concerne le genre ; la même constatation est faite pour la pression artérielle avec une prévalence de 39,4%. L'obésité abdominale est significativement correlée à la tension artérielle avec 211 sujets (23,4%) (P=0,01), au diabète avec 33 personnes (5,8%) avec un coefficient de corrélation significative (P=0,003) et au genre (P=0,000), avec une nette prédominance chez les femmes (47,9%VS 6,7%). Quant au rapport tour de taille/tour de hanche (RTH) on note une association positive avec le diabète (P<0,05). Par ailleurs, sur les 200 personnes obèses, 165 (82,5%) ont présenté un RTH supérieur à la normale et sur les 258 personnes en surchage pondérale, 185 (71,7%) participants ont eu un RTH anormal (P<0,001). En outre, dans nos résultats préliminaires sur les habitudes alimentaires, nous avons constaté une faible consommation des fruits et des légumes et une surconsommation des sucres rapides (résultats non soumis dans cet article).

**Tableau 1:** Tableau descriptif de la population étudiée (exprimé en moyenne et écart-type)

Variables		Moyenne	Ecart-type	P	Effectifs
	Sexe				
**Âge**	Féminin	39,16	11,33	0,28	902
	Masculin	40,01	12,07		
**Poids**	Féminin	66,25	14,88	0,06	902
	Masculin	65,58	11,59		
**Tour de taille**	Féminin	92,85	17,36	0,000	902
	Masculin	84,11	13,20		
**Tour de Hanche**	Féminin	102,04	16,61	0,000	902
	Masculin	93,39	12,49		
**IMC**	Féminin	27,52	6,14	0,000	902
	Masculin	24,45	4,16		
**TT/TH**	Féminin	0,909	0,07	0,12	902
	Masculin	0,901	0,08		
**PA systolique**	Féminin	129,95	23,22	0,8	902
	Masculin	130,77	19,47		
**PA diastolique**	Féminin	70,83	20,56	0,02	902
	Masculin	73,94	18,90		
**Temps assis ou couché/jour**	Féminin	15,81	4,719	0,000	902
	Masculin	13,94	4,717		
Glycémie à Jeun*	Féminin	1,02	0,33	0,11	567
	Masculin	1,01	0,53		

* Glycémie prélevée chez 567 individus, **IMC** : Indice de Masse Corporelle **TT/TH** : tour de taille / tour de hanche **PA** : Pression Artérielle

**Tableau 2:** Facteurs de risque cardiovasculaires de la population étudiée en fonction de l’indice de masse corporelle (IMC) ^a^

Variables	IMC<18,5 kg/m^2^ (N=37)	18,5<IMC<30 kg/m^2^ (N=665)	IMC>30 kg/m^2^ (N=200)
Féminin	2,4***	40	17,4***
Masculin	1,7	33,7	4,8
Poids (kg)	44,8 (5,7) ***	62,4 (8,5)	84,2 (11,8) ***
Taille (cm)	161,5 (9,0)	160,2 (8,2)	156,3 (8,5)
Tour de taille (cm)	72,6 (13,0) ***	85,62 (13,6)	104,8 (14,9) ***
Tour de hanche (cm)	81,0 (14,7) ***	95,0 (12,9)	113,6 (13,7) ***
RTH	0,90 (0,1)	0,90 (0,07)	0,92 (0,07) ***
Tabagisme %	0,9	12,4	1,4 *
Alcoolisme %	0,0	0,2	0,0
** IAPR %**	2,4	54,4	16,9
Durée inactif/jour	14,70 (4,73)	14,77 (4,79)	16,09 (4,72) ***
** Hypertension %**	1	27,9	10,4*
TA systolique (mmHg)	124,8 (22,3)	128,8 (20,7)	136,2 (23,9) ***
TA diastolique (mmHg)	69,7 (14,5)	73,3 (18,3)	68,3 (24,8) **
	**N= 28**	**N=399**	**N=140**
** Glycémie (g/l)** ^b^	1,08 (0,71)	0,98 (0,41)	1,09 (0,44) **
HGJ (%) ^b^	0,5	4,4	3,2**
Diabète (%) ^b^	0,5	4,6	3,4**

**RTH** : rapport tour de taille/ tour de hanche ; **IAPR** : Inactivité physique régulière ; **TA** : tension artérielle ; **HGJ** : hyperglycémie à Jeun. **a** : exprimée en moyenne (écart-type) et pourcentage (%) ; **b** : Glycémie prélevée chez 567 participants ;

* P<0,05 ;

** P<0,01 ;

*** P< 0,001

## Discussion

La présente étude démontre la coexistence, au sein de la population de l'île d'Anjouan, de la sous-nutrition et de la surnutrition, confirmant ainsi la présence de la double charge nutritionnelle aux Comores. La présence simultanée de ces deux états nutritionnels chez les adultes en absence de guerre et de famine est surprenante. En effet, elle reflète d'une part, une malnutrition infanto-juvénile et maternelle et de l'autre, une transition nutritionnelle résultant d'une modification des habitudes alimentaires et du mode de vie due à une surconsommation en carbohydrates et en graisses animales, une faible consommation en fibres [3,17,18] et une sédentarité sévère [8]. Cette étude fait état de 4,1% d'individus en état de maigreur, de 28,6% de sujets en surpoids et de 22,2% d'obèses. La courbe d'évolution de l'insuffisance pondérale chez les enfants de moins de 5 ans est passée de 13,9% en 1991 à 15,3% en 2012 avec des variations non négligeables allant jusqu'à 26,3% en 2000 [12], ce qui, éventuellement, explique la présence du sous poids chez les adultes. De plus dans la même période, 30,1% de ces mêmes enfants souffraient d'un retard de croissance [12]. En 2011, l'enquête STEPwise a reporté des taux de 28,9% et de 16,6%, respectivement, pour le surpoids et l'obésité sur l'île d'Anjouan [19]; tandis qu'au niveau national les prévalences étaient de 25,9% et 13,5% [11], on observe nettement une augmentation de l'obésité au sein de cette population. A ces dernières, s'associe une prévalence élevée des pathologies chroniques (hypertension, diabète) non transmissibles, chez les sujets en surcharge pondérale (IMC>25 kg/m^2^), conditionnées par l'adoption d'un mode de vie décrit comme une « occidentalisation » des comportements [20,21]. La littérature évoque une prédominance féminine de l'obésité observée dans plusieurs populations, on cite le cas du Mexique avec une prévalence de 25% de l'obésité chez les femmes contre 15% chez les hommes [22], des Caraïbes anglophones avec 50% chez les femmes contre 25% chez les hommes [23] et le Maroc avec un taux de 20,9% chez les femmes contre 6% chez les hommes [24] et où la classe d'âge dominante chez les femmes, tout comme dans cette étude, se situe entre 35 et 49 ans [25] . Les résultats obtenus rejoignent cette tendance avec 17,4% chez les femmes contre 4,8% chez les hommes, à l'instar de ce qui a été observé en 2011 [11]. Cette différenciation entre les sexes a fait l'objet de plusieurs théories, notamment celle liée à l'apparition des menstruations qui s'accompagnent d'un changement hormonal favorisant l'accumulation des graisses corporelles chez les femmes [18]. A cela, s'ajoute la sédentarité qui est significativement fréquente chez les femmes (P<0,01), probablement due au facteur culturel qui les contraint à s'occuper des tâches ménagères ce qui leur laisse moins de temps pour des rôles comprenant des exercices physiques vigoureux et régulières [26], le nombre de grossesses et d'enfants [25].

En outre, on met également en cause les préjugés sociaux qui étiquettent le surpoids/obésité comme critères de beauté chez les femmes et d'aisance dans le ménage. Les sujets souffrant d'insuffisance pondérale (IMC<18,5 kg/m^2^) n'ont associé aucun facteur de risque cardiovasculaire contrairement à d'autres auteurs qui ont observé une association significative entre un faible IMC et une hyperglycémie [26]. Toutefois, D'autre part, les auteurs s'accordent sur le lien étroit entre le surpoids/l'obésité et le développement du diabète et de l'hypertension [27, 28]. L'adiposité centrale et/ou le rapport tour de taille/tour de hanche élevée sont en grande partie responsable de la résistance à l'insuline et de l'apparition du syndrome métabolique (obésité, intolérance au glucose, hypertension, hyperlipidémie, hyperuricémie et autres anomalies métaboliques [29]. Une étude réalisée au Bénin [30] a montré que l'obésité abdominale est positivement associée à une probabilité accrue de syndrome métabolique. L'obésité abdominale s'est également révélée être un facteur de risque important d'insuffisance cardiaque chez les adultes en Centre Afrique, où les adultes présentant un rapport tour de taille/tour de hanche élevé ont un risque accru d'insuffisance cardiaque [31]. Dans leur étude réalisée en Gambie, Van der Sande *et al*. ont retrouvé la même association positive entre l'adiposité abdominale et l'hypertension, l'hyperlipidémie et l'hyperuricémie [27]. Nos données rejoignent les auteurs cités précédemment; en effet, l'obésité abdominale est positivement corrélé avec le diabète (P=0,003) et l'hypertension (P=0,01) ; quant au RTH l'association positive ne concerne que le diabète (P=0,028). La consommation du tabac et d'alcool sont, comme l'indique la littérature, des facteurs décisifs dans l'apparition du diabète, des maladies cardiovasculaires et certains cancers. En 2011, 12,9% de la population comorienne étaient des consommateurs de tabac [11] avec une nette prédominance chez les hommes (23,8% VS 2%). On retrouve le même dynamisme dans notre étude (P=0,000) particulièrement dans la classe d'âge 50-64 ans (P=0,000). Par ailleurs, la consommation de tabac est positivement corrélée à l'obésité (tableau 2) et à l'hypertension (P=0,04). Baalwa *et al*. [26] ont montré une association positive entre la consommation de tabac et la prise de poids contrairement à Ba ML qui évoque l'inverse [32].

Les données obtenues concernant la consommation d'alcool peuvent sembler déconcertantes (0,2%) soit une personne sur les 902 participants, néanmoins ceci peut s'expliquer par la religion musulmane qui domine indubitablement dans le pays et qui interdit formellement la consommation de l'alcool. Les résultats du StepWISE corroborent les nôtres avec un taux de 0,9% au niveau national [11]. L'obésité, malgré ses risques, n'est pas toujours perçue comme un facteur de risque pour la santé. Bien inversement, la surcharge pondérale ainsi que l'inactivité physique sont considérés comme signe d'aisance et de prospérité [27]. Néanmoins, ils constituent des facteurs de risque majeurs dans la mortalité par maladies cardiovasculaires [10]. L'exemple des îles du sud-ouest de l'océan Indien est démonstratif de l'importance des facteurs de risque dans l'éclosion des maladies cardiovasculaires. Ces îles présentent de fortes disparités au plan des indicateurs démographiques et économiques. La mortalité par maladies cardiovasculaires est très élevée aux Seychelles (32 %), à Maurice (31 %) et à La Réunion (29 %), îles qui ont terminé leur transition épidémiologique. Cette mortalité par maladies cardiovasculaires reflète une haute prévalence des facteurs de risque ainsi que le vieillissement de la population [3]. A l'opposé, la mortalité par maladies cardiovasculaires dans les îles en phase de transition épidémiologique est basse: 15 % aux Comores, 18 % à Madagascar, cependant la prévalence des facteurs de risque est élevée dans ces 2 îles en ce qui concerne l'hypertension artérielle et le diabète, laissant présager une augmentation rapide de la mortalité par maladies cardiovasculaires [3]. Cependant, nos résultats sont purement descriptifs notamment en ce qui concerne la consommation de tabac et d'alcool qui n'ont pas été quantifié et qui nécessiterait une étude plus approfondie. Pareillement pour l'état nutritionnel où en plus des paramètres anthropométriques, il serait considérable de mettre en perspective une étude clinique pour évaluer le taux de cholestérolémie au sein de cette même population.

## Conclusion

L'Afrique Subsaharienne n'en a malheureusement pas fini avec les maladies infectieuses, avec 53% des décès survenant en bas âge, qu'il s'alourdit avec les décès causés par les maladies chroniques non transmissibles [19,30,31]. Ces dernières s'accroissent d'année en année, représentant un lourd fardeau et un véritable problème de santé publique pour un continent déjà bien atteint. La surcharge pondérale particulièrement abdominale et l'obésité sont des facteurs primordiaux dans l'apparition des pathologies chroniques, leur augmentation dans la population anjouanaise parallèlement au diabète et à l'hypertension est très alarmante, du fait qu'elles nous avisent sur une expansion de la mortalité par maladies non transmissibles dans un pays luttant contre la mortalité infantile (de moins de 5 ans) due aux pathologies infectieuses et aux carences nutritionnelles. Il est par conséquent urgent de mettre en place des stratégies efficientes afin d'atténuer leur progression.

### Etat des connaissances actuelles sur le sujet

- L'obésité représente un véritable problème de santé publique, vu sa progression croissante notamment dans les pays à faible ou à revenu moyen;
- L'obésité est un facteur de risque majeur dans le développement du diabète et des maladies cardiovasculaires;
- La surcharge pondérale, survient à un moment où les pays en développement luttent encore contre les carences nutritionnelles et les pathologies infectieuses, constituant pour ces derniers un double fardeau.

### Contribution de notre étude à la connaissance

- Etude de la prévalence de l'insuffisance pondérale, du surpoids et de l'obésité chez les adultes de l'île d'Anjouan;
- Nos résultats démontrent l'existence du double fardeau nutritionnel, ce qui d'une part, confirme la transition épidémiologique, et d'autre part soulève le problème non résolu des maladies carentielles aux Comores;
- Nos données prouvent la nécessité urgente d'une mise en place de stratégies de prévention ciblées.
